# Sampling Optimization and Crop Interface Effects on *Lygus lineolaris* Populations in Southeastern USA Cotton

**DOI:** 10.3390/insects13010088

**Published:** 2022-01-13

**Authors:** Seth J. Dorman, Sally V. Taylor, Sean Malone, Phillip M. Roberts, Jeremy K. Greene, Dominic D. Reisig, Ronald H. Smith, Alana L. Jacobson, Francis P. F. Reay-Jones, Silvana Paula-Moraes, Anders S. Huseth

**Affiliations:** 1 Department of Entomology and Plant Pathology, North Carolina State University, Raleigh, NC 27695, USA; 2 Forage Seed and Cereal Research Unit, U.S. Department of Agriculture-Agricultural Research Service (USDA-ARS), Corvallis, OR 97331, USA; 3 Department of Entomology, Virginia Tech, Tidewater Agricultural Research and Extension Center, Suffolk, VA 23437, USA; svtaylor@vt.edu (S.V.T.); smalone@vt.edu (S.M.); 4 Department of Entomology, University of Georgia Tifton Campus, Tifton, GA 31793, USA; proberts@uga.edu; 5 Department of Plant and Environmental Sciences, Edisto Research and Education Center, Clemson University, Blackville, SC 29817, USA; greene4@clemson.edu; 6 Department of Entomology and Plant Pathology, Vernon James Research and Extension Center, North Carolina State University, Plymouth, NC 27962, USA; ddreisig@ncsu.edu; 7 Department of Entomology and Plant Pathology, Auburn University, Auburn, AL 36849, USA; smithrh@auburn.edu (R.H.S.); alj0043@auburn.edu (A.L.J.); 8 Department of Plant and Environmental Sciences, Pee Dee Research and Education Center, Clemson University, Florence, SC 29501, USA; freayjo@clemson.edu; 9 Entomology and Nematology Department, West Florida Research and Education Center, University of Florida, Jay, FL 32565, USA; paula.moraes@ufl.edu

**Keywords:** *Gossypium hirsutum*, risk factors, sampling plan, scouting, tarnished plant bug

## Abstract

**Simple Summary:**

Tarnished plant bugs (Hemiptera: Miridae) are an important agricultural pest in cotton across the United States. Tarnished plant bugs reduce cotton yields and lower lint and seed quality by feeding on reproductive structures. Effective management of this pest requires timely insecticidal control when populations reach established economic thresholds. Reliable determination of the economic threshold in cotton depends on effective scouting. To evaluate the efficacy of current tarnished plant bug scouting strategies, we surveyed 120 commercial cotton fields across the southeastern USA to quantify (1) variation in tarnished plant bug populations across the production region, (2) evaluate current sampling plans for economic threshold determinations, and (3) examine landscape-scale risk factors associated with tarnished plant bug infestations in cotton. We observed the greatest variability in tarnished plant bug density at the field scale followed by within-field variation, emphasizing the importance of scouting individual fields. Additionally, we determined the sampling size needed for accurate threshold estimates for sweep net (8 sample units of 100 sweeps/sample) and drop cloth sampling (23 sampling units of 1.5 row-m/sample). Furthermore, tarnished plant bugs densities were positively related to the proportion of agriculture and double-crop winter wheat and soybeans and negatively related to contiguous cotton.

**Abstract:**

Tarnished plant bug, *Lygus lineolaris* (Hemiptera: Miridae), is an economically damaging pest in cotton production systems across the southern United States. We systematically scouted 120 commercial cotton fields across five southeastern states during susceptible growth stages in 2019 and 2020 to investigate sampling optimization and the effect of interface crop and landscape composition on *L. lineolaris* abundance. Variance component analysis determined field and within-field spatial scales, compared with agricultural district and state, accounted for more variation in *L. lineolaris* density using sweep net and drop cloth sampling. This result highlights the importance of field-level scouting efforts. Using within-field samples, a fixed-precision sampling plan determined 8 and 23 sampling units were needed to determine *L. lineolaris* population estimates with 0.25 precision for sweep net (100 sweeps per unit) and drop cloth (1.5 row-m per unit) sampling, respectively. A spatial Bayesian hierarchical model was developed to determine local landscape (<0.5 km from field edges) effects on *L. lineolaris* in cotton. The proportion of agricultural area and double-crop wheat and soybeans were positively associated with *L. lineolaris* density, and fields with more contiguous cotton areas negatively predicted *L. lineolaris* populations. These results will improve *L. lineolaris* monitoring programs and treatment management decisions in southeastern USA cotton.

## 1. Introduction

The spatial heterogeneity of arthropod pest populations in crop fields is a factor that complicates accurate density estimation and the use of economic thresholds. A wide range of biological and ecological processes influence variation in pest distribution, including dispersal ability, habitat selection, inter- and intra-species interactions, and aggregation behavior [1,2,3]. To address this challenge, estimating pest densities using systematic pest scouting has become a foundational decision support tool to anticipate economic damage in an integrated pest management (IPM) framework [4,5]. Unfortunately, determining the number of sampling units needed to make reliable population estimates with high precision can require a significant amount of effort depending on the spatial distribution of the pest and tolerance for crop injury [4]. To address aggregated distributions of pests, researchers have applied analytical approaches such as variance around the population mean or degree of spatial clustering to inform population estimates or distribution patterns in cultivated crops, respectively [6,7,8,9,10]. Moreover, frequency distribution models can be used to estimate the minimum sample size for accurate population estimates of arthropods [11]. Collectively, the deliberate improvement of sampling approaches will contribute to effective IPM implementation by enhancing the reliability of action thresholds.

For many pests with short dispersal distances, the local landscape composition adjacent to crops can be used to inform field-level risk and subsequent sampling effort during the growing season. Geospatial modeling that associates arthropod abundance with environmental predictors has provided ecological and behavioral insights about arthropod pests in many different agricultural crops [12,13,14,15,16,17,18]. These models have been used to develop biotic (habitat composition and configuration) and abiotic (climate) risk descriptors that help guide pest management efforts at multiple scales. However, these geospatial studies rarely connect landscape-level risk with sample optimization to generate a unified estimation of field-level risk.

In southeastern USA cotton production regions, the tarnished plant bug, *Lygus lineolaris* (Palisot de Beauvois) (Hemiptera: Miridae), is a common pest that has a unique association with both crop and non-crop hosts [18] and presents consistent challenges for researchers trying to quantify populations within the cotton crop itself. *Lygus lineolaris* is a polyphagous sap-feeding insect that feeds on cotton reproductive structures (terminals, squares, flowers, and small bolls) with piercing-sucking mouthparts and digestive enzymes that break down plant tissue [19,20,21]. *Lygus lineolaris* cycles through a predictable sequence of non-crop and crop hosts throughout the year [22,23]. In the southern USA, *L. lineolaris* colonization of cultivated hosts often occurs after primary weedy hosts senesce during the late spring to early summer [24]. Although many studies have examined host use at fine scales, limited research has investigated the sequential movement of *Lygus* species among cultivated crops during the growing season [18,24,25,26]. In Virginia and North Carolina, landscape factors contributing to the source–sink movement of *L. lineolaris* populations to commercial cotton fields have been studied [18,27]; however, these results have not been validated across the southeastern USA.

Many studies have optimized *L. lineolaris* sampling techniques in cotton [28] and developed economic thresholds for improved decision-making [29,30,31,32]. Core findings from these studies demonstrated that sampling bias exists between sweep net and drop cloth sampling techniques toward adult and nymphal densities, respectively [28,33]. As such, the current University Extension recommended economic thresholds for sweep net sampling (i.e., 8 *L. lineolaris* adults and nymphs per 100 sweep net samples in addition to square retention below 80 percent) are most effective during cotton squaring when *L. lineolaris* are immigrating into cotton fields, and a drop cloth economic threshold (i.e., 2.5 *L. lineolaris* adults and nymphs per 1.5 row-m) is recommended during cotton flowering to assess nymphal populations [28,29,30,31,34]. One challenge for applying these thresholds in commercial cotton is accounting for inconsistencies among observers when estimating the mean immature or adult *L. lineolaris* populations. To address this knowledge gap, a recent study focused on the application of large observational datasets to refine understanding of inter-observer repeatability when scouting *Lygus hesperus* (Knight) in commercial cotton fields [35]. Furthermore, Rosenheim [35] suggested that future studies could examine sources of sample variation within differently sized fields to understand the relative importance of sub-sample differences within cotton fields.

In this study, we intensively scouted georeferenced transects nested within commercial cotton fields across the southeastern USA to understand the sources of *L. lineolaris* population variation and to identify linkages between field-level estimates and landscape composition adjacent to fields. Our specific research objectives were to (1) evaluate *L. lineolaris* abundance variation in cotton at different spatial scales, (2) develop a fixed-precision sampling plan for *L. lineolaris* using sweep net and drop cloth sampling techniques during the squaring and bloom growth stage, respectively, and (3) validate local landscape risk factors of *L. lineolaris* adults moving into cotton. We hypothesized *L. lineolaris* abundance variation would be greatest at the field scale (i.e., variation among fields) based on dispersal biology of this pest [36] and prior work demonstrating stark differences in *L. lineolaris* abundance at fine spatial scales throughout the cotton growing season [18]. Additionally, we hypothesized that a higher abundance of early season cultivated crop hosts (e.g., small grains, corn) in the surrounding landscape would be positively related to *L. lineolaris* abundance in cotton 8,24,26].

## 2. Materials and Methods

### 2.1. Field Selection and L. lineolaris Sampling

**Figure 1:**
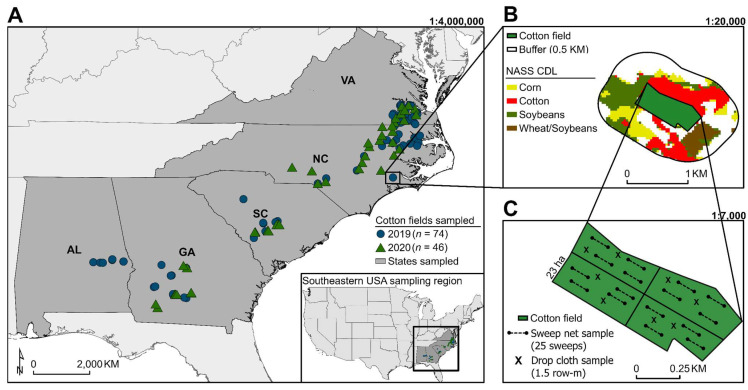
(**A**) Commercial cotton fields (*n* = 120) sampled across the southeastern USA in 2019 and 2020. (**B**) Local landscape data extraction using a 0.5 km buffer around field borders and the NASS CropScape raster data layer. (**C**) Within-field sampling scheme consisting of four 25 sweep net sampling units and two drop cloth (1.5 row-m) units per quadrant for sweep net and drop cloth sampling, respectively.

To survey *L. lineolaris* abundance across the southeastern USA cotton growing region, commercial cotton fields in five states (Alabama, Georgia, North Carolina, South Carolina, Virginia) were systematically sampled in 2019 and 2020, for a total of 74 and 46 unique fields in each year, respectively (*n* = 120 total fields) (Figure 1A). Fields were all located within the typical row crop production system found in the southeastern USA, and consisted of a range of crops that include cotton, corn, soybean, and small grains (Figure 1B). The mean area of sampled fields averaged 28.8 ha ± 34.2 SD. Each field was visited twice during the cotton growth stages susceptible to *L. lineolaris* feeding damage: one sample pre-bloom (when pre-floral buds or squares were present) and one sample post-bloom (within 3 weeks of >50% of the field in bloom). Fields were not sampled if producers sprayed pesticides (herbicide or insecticide) within 3 days of sampling or if inclement weather could affect insect density measurements [18,33].

Fields were divided into sampling quadrants for even sampling distribution across fields (Figure 1C). Spatial coordinates in decimal degrees were recorded for the specific sample location within each quadrant during the pre-bloom sampling event; we returned to the same location for the post-bloom sample. The sampling scheme was consistent for each quadrant, including sweep net and drop cloth sampling for *L. lineolaris* adult and nymphal densities. For sweep net sampling, four independent transects of 25-sweep samples (38 cm diameter) across one row were conducted in each quadrant (*n* = 100 sweep samples per quadrant; *n* = 400 total sweep samples per field) [28]. Four locations were randomly selected for drop cloth (91 × 76 cm) sampling between two rows within each quadrant (*n* = 16 total drop samples per field). Samples were taken at least 15 m from field borders to reduce edge effects [37]. Adult and nymphal densities were recorded for both sampling techniques, and count data were recorded by the unit of observation (one 25-sweep sample or one drop sample) for each quadrant nested within fields. *Lygus lineolaris* abundance variation during cotton squaring was analyzed using total adult and nymphal counts with sweep net sampling, and *L. lineolaris* abundance variation during cotton flowering was analyzed using drop cloth assessments.

### 2.2. Plant Injury Assessments

In addition to *L. lineolaris* sampling, plant injury was also recorded. For each field quadrant, square retention was recorded (pre-bloom and bloom) by checking the first fruiting position on the top five nodes (node = true leaf >25 mm diameter) of five random plants for square abscission due to *L. lineolaris* herbivory [38]. The number of abscised squares was recorded across the five nodes assessed per plant.

### 2.3. Landscape Data Extraction

To investigate potential crop interface effects on *L. lineolaris* populations in cotton, field digitization and landscape data extraction were conducted using ArcGIS software (Environmental Systems Research Institute ArcGIS Pro, Version 2.7.1, 2020, Redlands, CA, USA). Perimeters of sampled fields were first digitized to polygons. Buffers were then generated around field polygons with offset distances of 0.5 km from field edges. Land area of potential *L. lineolaris* source habitat within buffers was extracted using the National Agricultural Statistics Service CropScape raster data layers (NASS-CDL) at 30 m × 30 m resolution, corresponding to the appropriate sampling year (2019–2020) [39]. Potential source habitat of *L. lineolaris* populations in cotton, based on prior research, included (1) double-crop winter wheat and soybeans (hereafter, ‘double-crop wheat/soybeans’), (2) corn, (3) cotton, (4) forest, (5) peanuts, (6) soybeans, (7) total agricultural area (hereafter, ‘agricultural area’), and (8) wheat [18,22,23,24,26]. Double-crop wheat/soybeans refers to winter wheat and soybeans grown sequentially in the same year. Landscape predictors were reclassified into broad categories when appropriate (e.g., “evergreen forest,” “deciduous forest,” and “mixed forest” grouped into “forest”). The proportion of land area extracted for each source habitat type within-field buffers was tabulated using the total buffer area for each field (Figure 1B).

### 2.4. Statistical Analysis

#### 2.4.1. *Lygus lineolaris* Abundance Variation and Fixed Sampling Plan

All data analyses were performed in R (v4.0.4 R Core Team, 2020). To interpret and visualize variation in *L. lineolaris* abundance at different spatial scales, we performed variance component analysis (VCA) using the *VCA* package [40]. *Lygus lineolaris* abundance collected with sweep net and drop cloth sampling was used as the dependent variable for VCA analysis for squaring and bloom growth stages, respectively. Random effects variables or variance components consisted of year, state, district, field, and quadrant within field. Predictor variance components were modeled as random effects using analysis of variance (ANOVA) estimation with the anovaVCA function to determine percent of total variance in the dependent variable attributed to each variance component. Variance component analyses were performed separately for sweep net and drop cloth sampling techniques, and results were visualized using the varPlot function. To compare overall variability in *L. lineolaris* abundance using sweep net and drop cloth sampling, we compared coefficient of variation estimates for each sampling method using an asymptotic test [41].

We further analyzed within-field *L. lineolaris* variation to determine an appropriate fixed-precision sampling plan for reliable population estimates for sweep net sampling during squaring (*n* = 330 sample units) and drop cloth sampling during bloom (*n* = 439 sample units). Sample units were defined as 100 sweeps and 1.5 row-m estimates for sweep net and drop cloth sampling, respectively (i.e., one sample per field quadrant). To determine the appropriate sample size for each sampling method, we used the formula developed by Green [42], $N=am^{b-2}/D^{2}$, where a and b represent Taylor’s power law parameters calculated independently for each sampling technique, m represents the population mean, and D indicates the desired level of precision of 0.25 or within 25% of the mean (Sweep: a = 0.95 (0.87 to 1.03 95% CI), b = 1.11 (0.95 to 1.27 95% CI), R^2^ = 0.73; Drop: a = 1.08 (1.02 to 1.13 95% CI), b = 1.17 (1.05 to 1.30 95% CI), R^2^ = 0.82) [43,44]. Fixed-precision sampling plan results were validated using Resampling for Validation of Sampling Plans (RVSP 2.0) software [45], which resampled observations with replacement through 500 simulations to determine an average ($N_{avg}$), minimum ($N_{min}$), and maximum ($N_{max}$) sample size at a fixed-precision level for both sampling methods.

#### 2.4.2. Correlating *L. lineolaris* Density to Plant Injury

Plant injury data (percent square retention) collected in sampled quadrants were correlated with *L. lineolaris* abundance at the square (*L. lineolaris* per 100 sweeps) and bloom growth stage (*L. lineolaris* per 1.5 row-m) using a generalized linear mixed model (GLMM) in the *lme4* package [46]. Percent square retention was fitted to a Poisson distribution, and *L. lineolaris* abundance was analyzed as fixed effects with random effects intercepts for sampling quadrants nested within fields. The predict function in the *car* package [47] was used to predict plant injury using *L. lineolaris* density.

#### 2.4.3. Local Landscape Effects on *L. lineolaris* Abundance

Significant local landscape predictors of adult *L. lineolaris* immigrating into cotton fields were analyzed using the INLA (Integrated Nested Laplace Approximation) framework in the *inla* package [48]. A hierarchical Bayesian GLMM was developed using a stochastic partial differential equations (SPDE) approach to account for spatial autocorrelation in *L. lineolaris* abundance. Adult *L. lineolaris* using sweep net sampling at both growth stages was used as the response. For this spatial regression model, let $Y_{ta}$ be total *L. lineolaris* adults at coordinates s for field location i, where i=1,…,N. We assumed $Y_{ta}$ followed a Poisson distribution, such that $Y_{ta}\;\sim \;Poisson\left(\mu \left(s_{i}\right)\right)$ and *L. lineolaris* adults were modeled on the log scale as $\log\left(\mu \left(s_{i}\right)\right)=\alpha_{0}+\log\left(q_{i}\right)+X_{i}\beta +W\left(s_{i}\right)+u_{i}$, where $\log\left(q_{i}\right)$ is an offset term to adjust for the number of sampled quadrants per location i, $\alpha_{0}$ is the intercept, β is a vector of regression parameters, $X_{i}$ is a matrix of explanatory covariates, $W\left(s_{i}\right)$ represents spatial random effects, and $u_{i}$ is Gaussian random effects for crop growth stage during sampling. The residual $W(s_{i})$ accounting for spatial dependency among locations was approximated using the Gaussian Markov random field (GMRF) with correlations determined by the Matérn correlation function using the inla.spde2.matern function in the *inla* package. Diffuse priors were used for the model. The posterior distribution of covariates was considered significant if 95% confidence intervals (hereafter, ‘95% CI’) did not overlap zero. Predictor covariate ‘wheat’ was removed from the model to improve model fit determined by the deviance information criterion (DIC) value [49]. A cross-validation procedure was conducted to analyze model performance on “new” data by randomly splitting the dataset (*n* = 210) into training (80%) and testing data (20%) to make predictions. Training data were used to make predictions on “test” data using the inla.stack function and rerunning the model described above. Predictions and actual observations of the testing data were compared using linear regression in the *plm* package [50].

## 3. Results

### 3.1. Lygus lineolaris Abundance Variation and Fixed Sampling Plan

**Figure 2:**
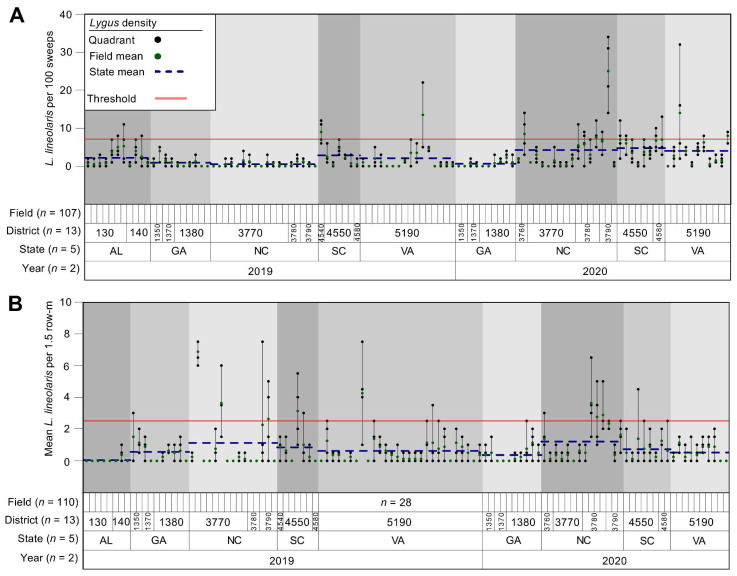
Variance component analysis (VCA) for sweep net sampling during cotton squaring (**A**) and drop cloth sampling during bloom (**B**). Colored points (green) represent field means, and dotted lines (blue) represent state means. Colored lines represent the sampling threshold for *Lygus lineolaris* economic injury using sweep net (8 *L. lineolaris* per 100 sweeps) and drop cloth sampling (2.5 *L. lineolaris* per 1.5 row-m).

*Lygus lineolaris* populations for sweep net sampling (per 100 sweeps) during the squaring growth stage pre-bloom averaged (mean ± SE) 2.2 ± 0.42, 1.2 ± 0.20, 3.0 ± 0.52, 3.9 ± 0.44, 3.8 ± 0.64 for Alabama, Georgia, North Carolina, South Carolina, and Virginia, respectively (*n* = 120 fields sampled). *Lygus lineolaris* adult and nymphal densities estimated using drop cloth sampling (per 1.5 row-m) during the bloom averaged 0.05 ± 0.05, 0.46 ± 0.10, 1.2 ± 0.30, 0.78 ± 0.20, 0.59 ± 0.12 for Alabama, Georgia, North Carolina, South Carolina, and Virginia, respectively (*n* = 109 fields sampled). Spatial variation in *L. lineolaris* abundance was greatest at the field scale across sampled regions of the southeastern USA compared with other spatial scales. Variation among sampled fields accounted for 44.5 and 45.1 percent of the variability in *L. lineolaris* abundance for sweep net and drop cloth sampling, respectively (Table 1, Figure 2). Similarly, within-field variability among sampled quadrants accounted for the second greatest percentage of variability in *L. lineolaris* abundance for both sampling methods at 31.8 and 37.0 percent variation for sweep net and drop cloth sampling (Table 1, Figure 2). Although the coefficient of variation estimates for drop cloth sampling at the bloom growth stage was numerically higher than sweep net sampling at squaring (Table 1), the variation in *L. lineolaris* counts among sampling methods was not significantly different (*p* = 0.15). Other predictors (i.e., sample year, state, agricultural district) accounted for considerably less variability in *L. lineolaris* estimates.

**Figure 3:**
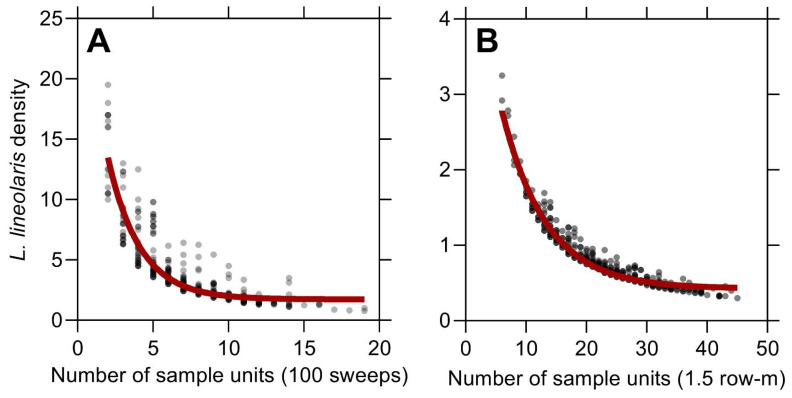
Validation of fixed-precision sampling plan by resampling with 500 simulations for sweep net sampling during cotton squaring (**A**) and drop cloth sampling (**B**) during the bloom growth stage. Red lines represent the stop line for the required sample size to estimate varying *L. lineolaris* population densities with 0.25 precision.

Results of the fixed sampling plan analysis found that 8.0 ± 2.9 SD ($N_{avg}$) sweep net sample units (each unit consisting of 100 sweep net samples) per field are required to obtain true population estimates of *L. lineolaris* at the squaring growth stage with a conservative level of precision (0.25). Mean *L. lineolaris* per sweep net sampling unit in the validation dataset was 2.9 ± 4.4 SD. Resampling validation indicated a minimum of 3.0 ($N_{min}$) and a maximum of 16.0 ($N_{max}$) sampling units were required to obtain *L. lineolaris* population estimates. Therefore, at least three sweep net sample units (300 sweeps) are required to determine if the *L. lineolaris* University Extension recommended economic threshold has been reached (Figure 3A). For drop cloth sampling during bloom, a sample size of 23.0 ± 7.3 SD ($N_{avg}$) drop cloth sampling units (per 1.5 row-m) are required to determine *L. lineolaris* population estimates. Mean *L. lineolaris* per drop cloth sampling unit was 0.7 ± 1.3 SD in the validation dataset. A minimum of 10.0 ($N_{min}$) and a maximum of 49.0 ($N_{max}$) samples are required to estimate *L. lineolaris* populations using drop cloth sampling at bloom for the desired precision level (Figure 3B). Therefore, at least 10 drop cloth samples are required to determine whether the *L. lineolaris* University Extension recommended economic threshold has been reached (Figure 3B).

**Figure 4:**
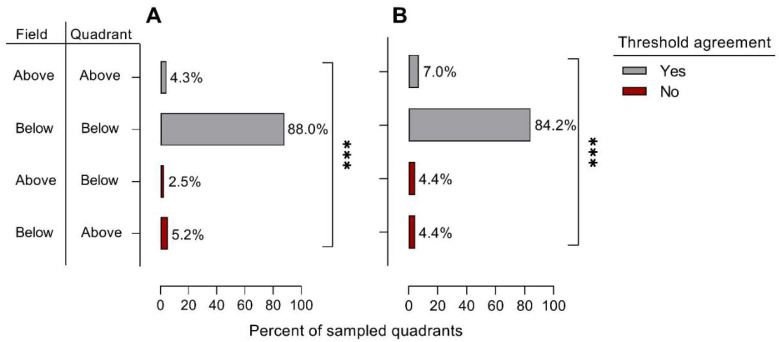
Percent of fields sampled with sweep net sampling during cotton squaring (**A**) and drop cloth sampling during bloom (**B**). Field and quadrant columns on the left represent *Lygus lineolaris* estimates above or below the economic threshold for respective sampling techniques. Floating bars highlighted in red indicate disagreement between quadrant and field-scale threshold determinations. Differences between threshold agreement and disagreement groups were analyzed using a chi-square independence test (*** *p* < 0.0001).

For field quadrant estimates of *L. lineolaris* using sweep net sampling during squaring (*n* = 330) and drop cloth sampling during bloom (*n* = 439), 88.0 and 84.2 percent of estimates at the quadrant and field scale were below the recommended economic threshold for sweep net and drop cloth sampling, respectively (Figure 4). The percent of sampled quadrants above the economic threshold in agreement with field-scale determinations of *L. lineolaris* abundance was 4.3 and 7.0 percent for sweep net and drop cloth sampling, respectively. The percent of quadrants in disagreement with field-scale determinations (above or below the economic threshold) was 7.7 and 8.8 percent of total observations for sweep net and drop cloth sampling, respectively. Therefore, when *L. lineolaris* populations were present at economically damaging levels for each sampling method, there was a 64.2 and 55.7 percent chance sampling a single quadrant would be inadequate for reliable management decisions for respective sampling techniques.

### 3.2. Correlating L. lineolaris Density to Plant Injury

**Figure 5:**
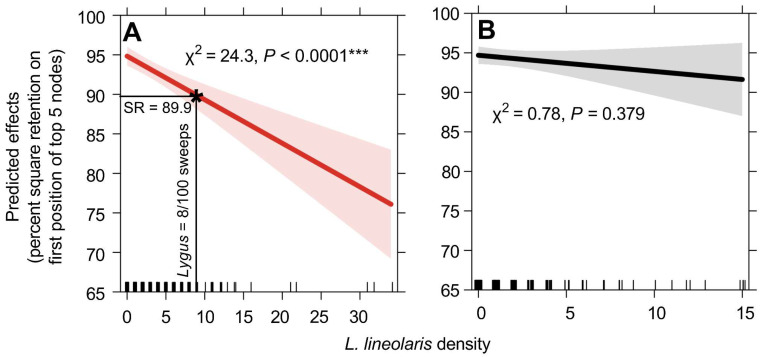
Model effects of percent square retention predicted by *Lygus lineolaris* density using sweep net sampling during the cotton squaring growth stage (**A**) and drop cloth sampling during the bloom growth stage (**B**).

Mean square retention across all states was high during cotton squaring in 2019 and 2020, averaging 93.4 and 93.7 percent retention each year, respectively. Similarly, square retention was high during the bloom growth stage, averaging 94.9 percent retention across both years of the study. *Lygus lineolaris* density was a significant predictor of square retention at the squaring growth stage ($\chi^{2}$ = 24.3, *p* < 0.0001; Figure 5A), but not at bloom ($\chi^{2}$ = 0.78, *p* = 0.38; Figure 5B). The current University Extension recommended economic threshold for sweep net sampling (8 *L. lineolaris* per 100 sweeps) predicted 89.9 percent square retention in sampled fields across the southeastern USA cotton growing region (Figure 5A). Moreover, field estimates of 24 *L. lineolaris* per 100 sweep samples (three times the current economic threshold) during squaring would be required to reduce square retention below 80 percent.

### 3.3. Local Landscape Effects on L. lineolaris Abundance

**Figure 6:**
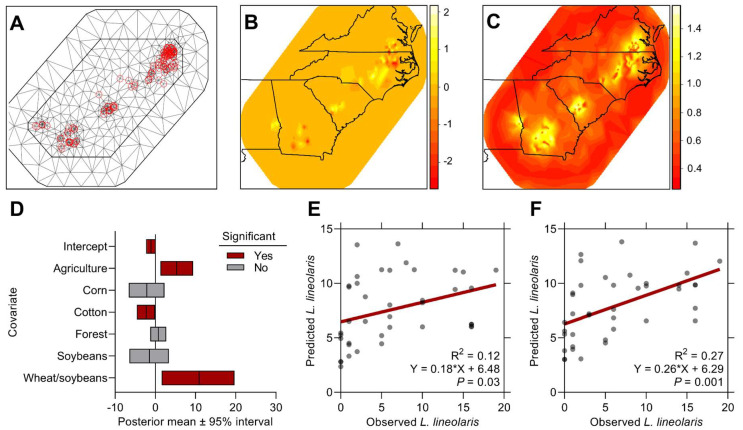
Mesh construction (**A**) for spatial random effects and the posterior mean (**B**) and standard deviation (**C**) of the spatial effect in the landscape model. Floating bars (**D**) represent predicted *Lygus lineolaris* (posterior mean ± 95% credible interval) of covariate predictors. Linear regression comparisons of observed and predicted *L. lineolaris* for the cross-validation procedure for the full model (**E**) and the proportion of agriculture covariate (**F**).

Crop interface covariates, including the proportion of agricultural land area, cotton, and double-crop wheat/soybeans, significantly predicted *L. lineolaris* abundance in commercial cotton across the sampled region (Table 2). Spatial random effects were included in the landscape model to account for spatial autocorrelation among sampled locations (Figure 6A), and the posterior mean (Figure 6B) and standard deviation (Figure 6C) of the spatial effect were visualized across the sampled area. The proportion of agricultural area ($\hat{\beta }$ = 5.32, 95% CI 1.25 to 9.38) and double-crop wheat/soybeans ($\hat{\beta }$ = 10.9, 95% CI 1.59 to 19.7) had a positive effect on *L. lineolaris* densities; cotton ($\hat{\beta }$ = −2.28, 95% CI −4.61 to −0.10) had a negative effect on *L. lineolaris* counts (Table 2, Figure 6D). Other covariates tested, including corn, were not significant. Model validation using linear regression analysis of observed and predicted estimates of testing data (20% of the total dataset) was significant for all predictors, although weakly correlated for the complete spatial model with all covariates (Y = 0.18X + 6.48; R^2^ = 0.12; *p* = 0.03; Figure 6E). When significant local landscape covariates were modeled independently, the proportion of agriculture was a marginally better predictor of *L. lineolaris* density in cotton (Y = 0.26X + 6.29; R^2^ = 0.27; *p* = 0.001; Figure 6F), compared with cotton (Y = 0.15X + 6.98; R^2^ = 0.17; *p* = 0.007) and double-crop wheat/soybeans (Y = 0.12X + 7.17; R^2^ = 0.11; *p* = 0.05).

## 4. Discussion

Spatial variation in *L. lineolaris* populations in cotton was most significant at the field scale. This finding agrees with Rosenheim [35], who documented a similar result for *L. hesperus* scouting data from commercial cotton fields in California. In both studies, scouting commercial cotton across a broad geographic extent underscored the value of field-specific scouting as a critical component of an effective integrated pest management program for *Lygus* species in the USA Cotton Belt. In this study, defined sampling quadrants explained the second-highest level of variability in *L. lineolaris* densities, suggesting a non-random, aggregated spatial distribution within fields. This result is not surprising, given previous work suggesting non-random, clustered distributions of closely related *Lygus* species in cotton [51,52] and other heteropteran species in field crop production systems [53,54,55,56]. In addition to field-specific scouting, intensive *L. lineolaris* sampling within fields is likely important to minimize the probability of false negative or positive assessments when making management decisions using economic thresholds. Moreover, sampling variation was numerically greater for drop cloth sampling during bloom. Considering *L. lineolaris* adults are highly mobile, the degree of spatial clustering is likely more severe for nymphal populations during cotton flowering [36,51]. Finally, we were surprised that state and district were not significant factors explaining variation in *L. lineolaris* populations, given the increases reported in North Carolina and Virginia [32], but not other states in the southeastern United States.

A fixed-precision sampling plan was developed for *L. lineolaris* in southeastern USA cotton based on sampling variation using sweep net and drop cloth sampling during the squaring and flowering growth stages, respectively. Although the recommended sampling size to estimate *L. lineolaris* populations in cotton may not be practical (32 sweep net samples of 25 sweeps per sample during squaring; 23 drop cloth samples during bloom), the minimum sampling size of 12 samples of 25 sweeps (300 total sweeps) pre-bloom and 10 drop cloth samples during flowering should be sufficient to detect populations above the recommended action thresholds (Figure 3). Furthermore, the proposed fixed sampling plan is likely more feasible and readily incorporated into existing pest management programs than more elaborate sampling plans for *L. lineolaris* in cotton, given the sporadic nature of this pest in the southeast and limited scouting efforts. We recommend the minimum sample size with sampling efforts partitioned systematically across fields for representative coverage and reliable threshold determinations of *L. lineolaris* populations above economically damaging levels.

Percent square retention assessments were high across the sampled region for the duration of the study. Similar to previous research, we found a significant negative relationship between *L. lineolaris* density and square retention pre-bloom [29,31]. Economic analyses for mid-southern *L. lineolaris* populations in cotton suggest >20 percent square abscission may result in reduced yield potential [29]. Considering populations across the southeastern USA generally peak in late July and August (during cotton flowering) [18], economically damaging populations may be rare pre-bloom, apart from exceptional situations. Furthermore, higher than current University Extension recommended threshold levels of *L. lineolaris* would be required to lower square retention below 80 percent, validating previous small-plot research suggesting a higher pre-bloom sweep net threshold may be warranted for *L. lineolaris* in the southeast (e.g., 12 to 16 per 100 sweeps) [34]. Additional small-plot and on-farm research is needed to refine economic thresholds in this region, given the differences in yield potential and emergence of new genetically engineered traits targeting *L. lineolaris* [57,58,59]. 

Crop habitats surrounding sampled fields described a significant amount of variation in *L. lineolaris* adult abundance in our study. However, the predictive power of the full landscape model and individual covariates to predict new observations was poor, as other studies observing local landscape composition effects on arthropod populations have shown [60]. Therefore, it is logical to assume local landscape features can contribute to yield-reducing outbreaks of *L. lineolaris* in cotton, but these scenarios are difficult to predict with small observational datasets such as the one used in this study [35,61]. The proportion of agriculture surrounding commercial cotton was the best landscape predictor of *L. lineolaris* tested, which confirms prior work documenting an association between *L. lineolaris* abundance and landscape composition in Virginia’s cotton agroecosystems [18]. Furthermore, this result agrees with extensive research into landscape simplification in agroecosystems and cropland dominance effects on increasing arthropod pest populations [12,17,62].

Double-crop winter wheat and soybeans was also a significant source of *L. lineolaris*. This crop rotation sequence may provide a continual source habitat of *Lygus* immigrants into a sink crop, such as cotton. This result also agrees with previous work documenting greater *Lygu*s populations in cotton near double-crop wheat/soybeans during the onset of cotton flowering [18] and high populations of *L. lineolaris* in soybeans [22]. On average, we sampled 3.0 ± 0.70 SE *L. lineolaris* per 100 sweeps in states with more double-crop wheat/soybeans cropland area (3.7 percent in NC, SC, and VA; double-crop wheat/soybeans: cotton ratio = 0.80). In contrast, states with lower double-crop wheat/soybeans (0.7 percent in AL and GA; double-crop wheat/soybeans: cotton ratio = 0.05), averaged 1.4 ± 0.41 SE *L. lineolaris* adults. Future research should investigate the source–sink effects of double-crop wheat/soybeans and cotton on *L. lineolaris* population dynamics by surveying both “source” and “sink” crops during the susceptible growth stages of cotton development. 

The proportion of cotton area surrounding sampled fields was negatively related to *L. lineolaris* abundance. Prior research has demonstrated a similar result for closely related *Lygus* species (*L. hesperus*) in cotton-dominant cropping systems [16,63,64]. We agree with previous studies that suggest this negative association between *Lygus* density and increasing cotton land area is likely explained by cotton’s reduced attractiveness compared with a more favorable habitat combined with strong flight capability of *L. lineolaris* and generally lower *Lygus* density in cotton compared with preferred hosts (e.g., flowering weeds) [25,51,64]. This putative habitat preference is supported by known differences in nutritional quality and reproductive potential of *L. lineolaris* with a generation time of 30 and 43 days (26.5 °C) on preferred weedy hosts and cotton, respectively [65].

The proportion of corn area was not a significant predictor of early-season *L. lineolaris* adults in cotton, contradicting previous research documenting a relationship between corn and *L. lineolaris* abundance in cotton [18,24]. Considering the broad sampling area of cotton in the present study with five states represented, we conclude that the corn area is an inconsistent predictor of *L. lineolaris* in southeastern cotton.

## 5. Conclusions

Results of this study highlight the heterogeneity of *L. lineolaris* populations within and among cotton fields in the southeastern USA. Using this regional survey, we generated new evidence that *L. lineolaris* is present at economically relevant levels across multiple states in the production region. This result is important because historically, this insect was considered a minor pest of cotton in the southeast. To address this emerging issue, we deployed a widespread, systematic sampling design to characterize the extent and intensity of this pest across different cotton agroecosystems in five states. At the field level, we found that the number of recommended sampling stops per field may need to increase to maximize the accuracy of population estimates. Results also supported the idea that some landscape features adjacent to cotton may relate to higher *L. lineolaris* infestations; however, developing an accurate risk prediction framework may demand a larger observational dataset that includes more fields across many production seasons. Collectively, this study is a step toward an improved *L. lineolaris* sampling strategy that unifies traditional threshold-based IPM with landscape ecology to reveal additional perspectives on pest risk in large-scale row crop agriculture.

## Figures and Tables

**Table 1:** Variance component analysis for *Lygus lineolaris* density (adults and nymphs) for sweep net sampling during the square growth stage and drop cloth sampling during the bloom growth stage. Sweep net samples (400 total sweeps) were collected in each field at each of four quadrant locations (4 independent 25 sweep sampling units summed per quadrant) and four drop cloth observations per field (two drop sampling units averaged per quadrant).

Technique	Source	df	VC ^a^	% Variation ^b^	CV ^c^
Sweep net (100 sweeps)	Year	1	1.94	9.01	0.48
	State	-	0	-	-
	District	8	2.40	11.1	0.53
	Field	94	9.58	44.5	1.06
	Field:quadrant	292	6.85	31.8	0.90
	Error	3	0.79	3.67	0.30
Drop cloth (1.5 row-m)	Year	1	314.1	1.15	34.6
	State	4	2725.3	9.98	10.2
	District	7	1846.3	6.76	84.0
	Field	66	12,310.8	45.1	216.8
	Field:quadrant	236	10,098.9	37.0	196.4
	Error	-	0	-	-

^a^ Negative variance component (VC) estimates set to zero; ^b^ percent of total variation in *L. lineolaris* density for each respective source; ^c^ coefficient of variation (CV).

**Table 2:** Local landscape model posterior mean estimates and 95% confidence intervals for covariate predictors of *Lygus lineolaris* density in commercial cotton fields.

Covariate	Posterior Mean	95% CI	Significant
Intercept	−1.08	[−2.09, −0.09]	Yes
Agriculture	5.32	[1.25, 9.38]	Yes
Corn	−2.12	[−6.61, 2.27]	No
Cotton	−2.28	[−4.61, −0.10]	Yes
Forest	0.74	[−1.23, 2.73]	No
Soybeans	−1.47	[−6.45, 3.35]	No
Double-crop wheat/soybeans	10.9	[1.59, 19.7]	Yes

## Data Availability

The data presented in this study are available upon request to the corresponding authors.
